# A Study of the Optimal Logic Combinations of RO-Based PUFs on FPGAs to Maximize Identifiability

**DOI:** 10.3390/s24237747

**Published:** 2024-12-04

**Authors:** Raúl Aparicio-Téllez, Miguel Garcia-Bosque, Guillermo Díez-Señorans, Francisco Aznar, Santiago Celma

**Affiliations:** 1 Group of Electronic Design (GDE), Aragón Institute of Engeneering Research (I3A), University of Zaragoza, 50009 Zaragoza, Spain; 2 Electronic Design Group, Aragón Institute of Engineering Research, Centro Universitario de la Defensa (CUD), 50090 Zaragoza, Spain

**Keywords:** authentication, hardware security, physically unclonable function, ring oscillator, sensor node, wireless sensor network

## Abstract

One of the challenges that wireless sensor networks (WSNs) need to address is achieving security and privacy while keeping low power consumption at sensor nodes. Physically unclonable functions (PUFs) offer a challenge–response functionality that leverages the inherent variations in the manufacturing process of a device, making them an optimal solution for sensor node authentication in WSNs. Thus, identifiability is the fundamental property of any PUF. Consequently, it is necessary to design structures that optimize the PUF in terms of identifiability. This work studies different architectures of oscillators to analyze which ones exhibit the best properties to construct a RO-based PUF. For this purpose, Generalized Galois Ring Oscillators (GenGAROs) are used. A GenGARO is a novel type of oscillator formed by a combination of up to two input logical operations connected in cascade, where one input is the output of the previous operation and the other is the feedback signal. GenGAROs include some previously proposed oscillators as well as many new oscillator designs. Thus, the architecture of GenGAROs is analyzed to implement a GenGARO-PUF on an Artix-FPGA. With this purpose, an exhaustive study of logical operation combinations that optimize PUF performance in terms of identifiability has been conducted. From this, it has been observed that certain logic gates in specific positions within the oscillator contribute to constructing a PUF with good properties, and by applying certain constraints, any oscillator generated with these constraints can be used to construct a PUF with an equal error rate on the order of or below $10^{-11}$ using 100-bit responses. As a result, a design methodology for FPGA-based RO-PUFs has been developed, enabling the generation of multiple PUF primitives with high identifiability that other designers could exploit to implement RO-based PUFs with good properties.

## 1. Introduction

Wireless sensor networks (WSNs) have gained particular relevance in recent years with the growth of the Internet of Things (IoT) due to their ability to exchange information while meeting low-cost and low-power-consumption requirements. WSNs consist of a network of sensor nodes that detect different types of physical data from the environment. These networks have been demonstrated to have applications in monitoring, tracking, and control in different fields such as medical, military, and environmental monitoring. In these environments where the exchanged information must be protected, it is essential to ensure that the sensor nodes participating in the WSN are properly authenticated [1,2]. However, proposed authentication solutions must also align with the inherent characteristics of WSNs and particularly low energy consumption.

Therefore, it is evident that one of the main risks that must be addressed in the field of hardware security is authentication. Typically, sensor nodes have restricted computational and memory resources, which significantly complicates protection against different types of attacks. To address this issue, currently, different Trusted Platform Modules (TPMs) are used for secure storage of cryptographic keys. However, while these are comprehensive solutions, they involve high costs and do not consider physical attacks [3].

Physically unclonable functions (PUFs) exploit the inherent variability of device manufacturing processes to generate binary sequences that uniquely authenticate devices [3,4,5,6]. In this way, given a certain challenge, a PUF generates a response which can be seen as the fingerprint of the device [7], a promising solution for secure device authentication [8] and key generation and storage [9]. In this regard, several PUF-based authentication schemes have already been proposed and analyzed, showing good performance and suitability in authentication of IoT devices [10,11,12].

RO-PUFs have been widely used because of their good performance and simple implementation in FPGAs [13]. Due to variations in the manufacturing process of devices, two identical ROs implemented in the same device oscillate at slightly different frequencies. An RO-PUF operates by comparing frequencies of identical ROs in pairs, so that the output bit depends on which RO oscillates faster [14]. However, RO-PUFs have also been demonstrated to be vulnerable against modeling attacks [15], partly caused by the spatial correlation of ROs in the FPGA [16,17]. To improve the performance of the RO-PUF, other types of oscillators have already been proposed and analyzed as PUF primitives, including configurable ring oscillators (CROs) [18,19], transient effect ring oscillators (TEROs) [20], self-timed ring oscillators (STROs) [21], Fibonacci ring oscillators (FIROs) [22], Galois ring oscillators (GAROs) [23], and generalized Galois ring oscillators (GenGAROs) [24,25], a novel type of oscillator consisting of a certain combination of up to two input logic gates in cascade, which is the focus of this work.

GenGAROs present an optimal approach for analyzing and proposing novel types of RO-based PUFs, as they include some previously proposed oscillators along with many new configurations of oscillators. In this work, an exhaustive study of the logic combinations of gates of GenGAROs is conducted with the objective of identifying new RO-based PUF primitives which can be used to create novel types of PUFs optimized for identifiability, which is the main property for sensor node authentication applications. Thus, the primary contribution of this work is the creation of a new design methodology for FPGA-based RO-PUFs, enabling the generation of multiple PUF primitives with high identifiability that other designers can leverage to implement RO-based PUFs with good properties.

This paper is organized as follows: Section 2 introduces GenGAROs and presents some key metrics of PUFs; Section 3 shows the optimization process followed to determine which GenGAROs present better properties; Section 4 analyzes the quality of several weak PUFs based on these GenGAROs; finally, conclusions are drawn in Section 5.

## 2. Background

### 2.1. GenGARO-PUF

**Figure 1:**
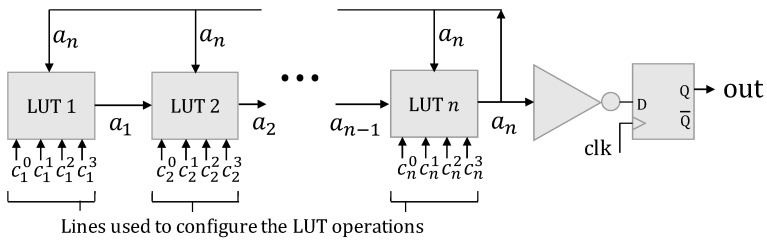
GenGARO architecture. $a_{n}$ refers to the feedback signal and ($c_{i}^{0},c_{i}^{1},c_{i}^{2},c_{i}^{3}$) to the configurations lines of LUT *i*.

In [24], the authors introduced the concept of generalized GARO-PUF (GenGARO-PUF). A GenGARO is a novel type of oscillator formed by a set of logical operations connected in cascade. Each operation takes as input the feedback signal and the output of the previous operation (Figure 1). In the first logic operation, as there is only one input, only a delay ($a_{1}=a_{n}$) or negation operation ($a_{1}=\bar{a_{n}}$) can be implemented. Throughout this work, the logic gate that performs the “delay” operation ($a_{1}=a_{n}$) will be referred as the “DEL” gate. As each LUT can be configured with a specific logic operation, this architecture of oscillator includes some of the previously proposed oscillators such as ROs (using only NOT operations) and GAROs (using XOR and NOT), but also many other additional oscillators. In previous work, it was observed that these structures presented low spatial correlation and good uniqueness and reproducibility compared to the spatial correlation of ROs [16].

GenGAROs also offer a higher number of possible oscillators compared to GAROs, thereby allowing the construction of a larger number of potential PUFs. Furthermore, uniqueness and reproducibility seemed to improve with the length of the oscillator. However, a configuration of GenGARO which outperformed the RO-PUF in terms of identifiability could not be obtained. Here, the word “configuration” refers to a certain combination of logic operations. Additionally, the authors opened the door to studying novel configurations of larger GenGAROs which simultaneously combine the properties of low spatial correlation and high uniqueness. In this work, the architecture of GenGAROs is analyzed to find which are the optimal configurations to construct a PUF with high identifiability.

### 2.2. PUF Evaluation Metrics

A PUF must generate a stable response over time (reproducibility). Furthermore, responses generated by different devices must be different (uniqueness). Typically, the intra-Hamming distance (*HD*) is used to measure the reproducibility of the PUF, while the inter-*HD* assesses uniqueness. Ideally, the intra-*HD* should be 0% while the inter-*HD* should be 50% on average.

**Figure 2:**
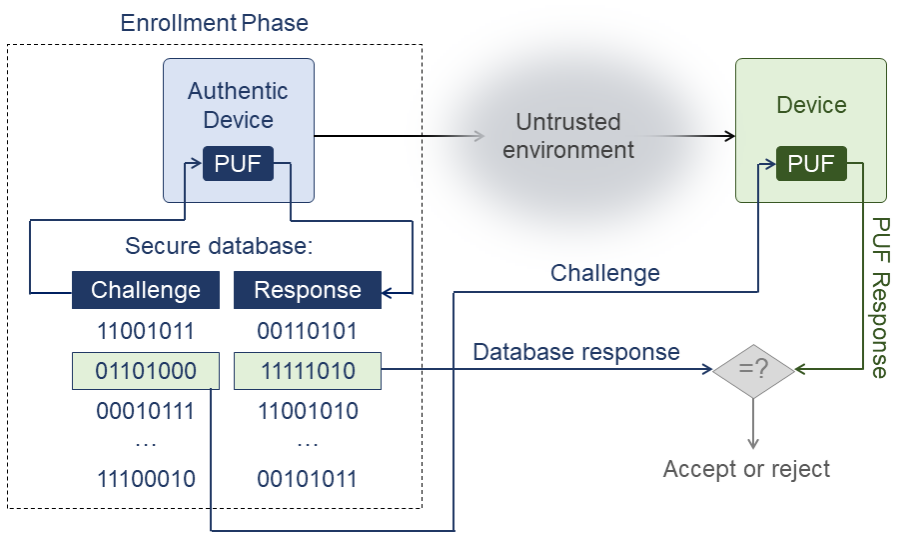
PUF-based authentication [26].

As previously discussed, one of the main applications of PUFs is device authentication. Typically, in a PUF-based authentication scheme, the PUF response is compared with a list of previously saved responses (Figure 2), so that if there is a response with an *HD* equal to or less than a an identification threshold $t_{id}$, the device is authenticated.

To quantify the identifiability of the PUF, the equal error rate (*EER*) [7] is used as figure of merit as it gives the probability of an identification attempt to result in a false rejection (false rejection rate, *FRR*) or false acceptance(false acceptance rate, *FAR*):

(1) $$\mathrm{EER}=\max\{\mathrm{FAR}\left(t_{\mathrm{EER}}\right),\mathrm{FRR}\left(t_{\mathrm{EER}}\right)\},$$

where $t_{\mathrm{EER}}$ is the corresponding identification threshold, defined as

(2) $$t_{EER}={\mathrm{argmin}}_{t}\left\{\max\left\{FAR\left(t_{id}\right),FRR\left(t_{id}\right)\right\}\right\}.$$

This parameter is especially interesting as it simultaneously combines the properties of uniqueness and reproducibility. Therefore, a low *EER* means a high reproducibility and uniqueness.

## 3. Methodology

### 3.1. FPGA Implementation

**Figure 3:**
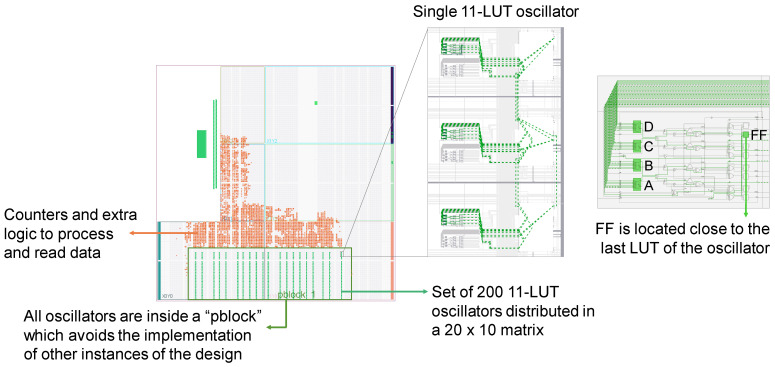
GenGARO-PUF implementation in Artix-7 FPGA.

In this work, a set of 200 11-LUT GenGAROs was implemented in 40 Artix-7 FPGAs (Figure 3). Artix-7 FPGAs have two slices per configurable logic block (CLB). Each slice contains 4 six-input LUTs, 8 flip-flops (FFs), and 4 multiplexers (MUXs). The 11-LUT GenGAROs are used because this allows each oscillator to be implemented using only three slices of the FPGA. Furthermore, as the number of LUTs of the oscillators increases, a higher number of configurations of oscillators can be evaluated. However, it must be noted that implementing oscillators with a high number of LUTs would result in a large consumption of the FPGA area. Each oscillator was implemented in LUT A, B, C, and D of slice $X_{i}Y_{j}$; LUT A, B, C, and D of slice $X_{i}Y_{j+1}$; and LUT A, B, and C of slice $X_{i}Y_{j+2}$; while an extra inverter was implemented in LUT D of slice $X_{i}Y_{j+2}$ to minimize possible frequency couplings.

An FF was also implemented to measure the bias of the signal of the oscillators. The FF was placed close to the extra inverter to prevent signal degradation. Additionally, locations and routing were carefully selected so that all oscillators were identical.

### 3.2. Experimental Scheme and Data Collection Process

The data collection process was performed in parallel so that every measurement was performed simultaneously in all FPGAs under the same voltage and temperature conditions. Furthermore, once the bitfile was generated, oscillators were externally reconfigured without the need to change the bitfile each time a novel configuration of GenGAROs was implemented.

To estimate the bias, $10^{6}$ samples were taken using a 1 MHz clock reference signal. These parameter choices stem from the compromise of achieving a reasonable authentication time while also avoiding the use of high-frequency reference clocks, as previous works have shown that high-frequency clocks lead to correlations between biases of oscillators implemented in the same FPGA [27].

The challenge with regard to the PUFs was the configuration of the logic gates. To obtain the PUF response, biases were compared in pairs using the non-overlapping comparison topology, ensuring that no oscillator was repeated in each comparison, thus avoiding correlations between successive bits of the response. Regarding oscillator comparison, one issue with the conventional RO-PUF is the spatial frequency correlation among oscillators. This makes it critical to compare nearby oscillators to obtain the PUF response. As the bias of GenGAROs do not present spatial correlation within the FPGA [24], this would eliminate the need to compare oscillators in a specific order, although close oscillators were compared in this work.

### 3.3. Optimization Process Description

Using a logic simulation, it was observed that approximately only one-third of all configurations lead to an oscillating output, while the other oscillators present a “logic fixed point”, i.e., stable output. Therefore, using 11-LUT GenGAROs, there are approximately $2\times 8^{10}/3≃7\times 10^{8}$ possible configurations that could be used to generate the PUF response. To determine which are the best configurations to construct a PUF with optimal properties, several restrictions were applied to study which combination of logic operations most contributed to obtaining a PUF with high identifiability.

**Figure 4:**
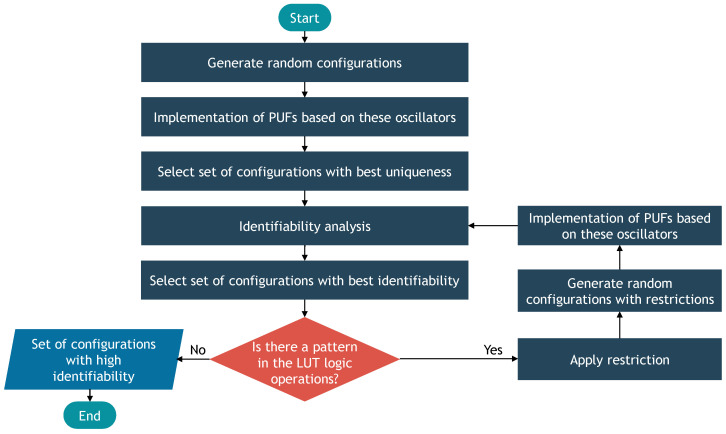
Optimization process.

This process is summarized in Figure 4.

## 4. Results

Following the optimization process, several constraints on LUT operations were applied to analyze the relation between logical operations of GenGAROs and PUF quality.

### 4.1. Without Restrictions

#### 4.1.1. Uniqueness

**Figure 5:**
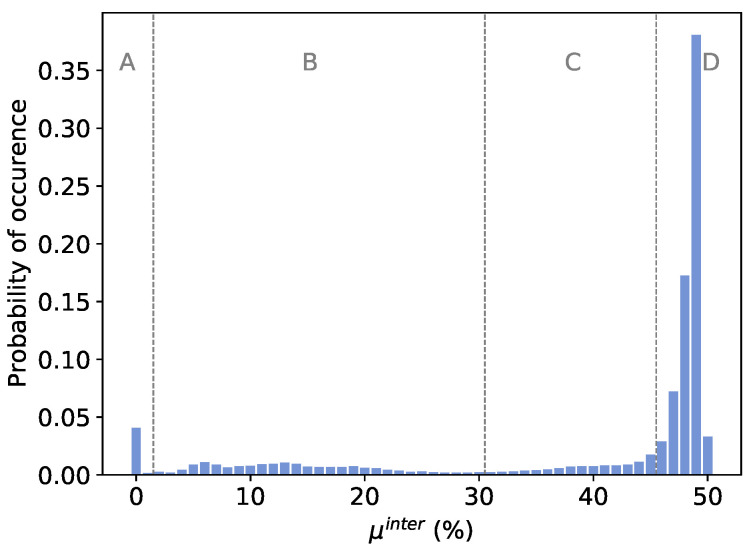
Average inter-*HD*s of 72,000 GenGARO-PUFs, each one implemented in 40 FPGAs using random configurations. A, B, C and D refer to each one of the four intervals.

Firstly, as the total number of possible configurations is too large to be analyzed in a reasonable time, a set of 72,000 configurations was randomly selected. Once the biases of the oscillators for each configuration were measured, the responses that would be obtained when using these oscillators to construct a GenGARO-PUF were simulated. In Figure 5, a histogram of the average inter-*HD*s ($\mu^{\mathrm{inter}}$) obtained for each configuration is shown.

As can be seen, the majority of the configurations (70.5%) present a high uniqueness, with $\mu^{\mathrm{inter}}\geq 45\%$. To analyze the combination of logic gates with the best uniqueness, the histogram was divided into four intervals: A, B, C, and D. Furthermore, the logic operations of ${\mathrm{LUT}}_{1}$ to ${\mathrm{LUT}}_{8}$ do not seem to have an impact on the uniqueness of the responses. Therefore, the study was centered on the last positions of the oscillators (${\mathrm{LUT}}_{9}$ to ${\mathrm{LUT}}_{11}$). In Table 1, the number of configurations f(x) presenting a certain logic gate in each position and interval is shown:

- $A=\{f\left(x\right)|\mu^{\mathrm{inter}}\left(f\left(x\right)\right)<1\%\}$: A total of 4.08% of the configurations present $\mu^{\mathrm{inter}}<1\%$. Those configurations with bad uniqueness usually present a NOT in ${\mathrm{LUT}}_{10}$ and a NAND in ${\mathrm{LUT}}_{11}$.
- $B=\{f\left(x\right)|\mu^{\mathrm{inter}}\left(f\left(x\right)\right)\in \left[1,30\right)\%\}$: A total of 16.76% of the configurations present are in this interval. These configurations usually present a DEL in ${\mathrm{LUT}}_{11}$.
- $C=\{f\left(x\right)|\mu^{\mathrm{inter}}\left(f\left(x\right)\right)\in \left[30,45\right)\%\}$: A total of 8.66% of the configurations present are in this interval. These configurations usually present a NOT in ${\mathrm{LUT}}_{10}$ and XOR, XNOR, NAND, or NOR gates in ${\mathrm{LUT}}_{11}$.
- $D=\{f\left(x\right)|\mu^{\mathrm{inter}}\left(f\left(x\right)\right)\geq 45\%\}$: A total of 70.5% of the configurations are in this interval. Those configurations with high uniqueness usually present AND/OR gates in ${\mathrm{LUT}}_{10}$, while they do not present NOT/NAND/NOR gates in this position. Furthermore, they usually present NAND/NOR gates in ${\mathrm{LUT}}_{11}$, while they do not present DEL/AND/OR gates in this position.

#### 4.1.2. Reproducibility

**Figure 6:**
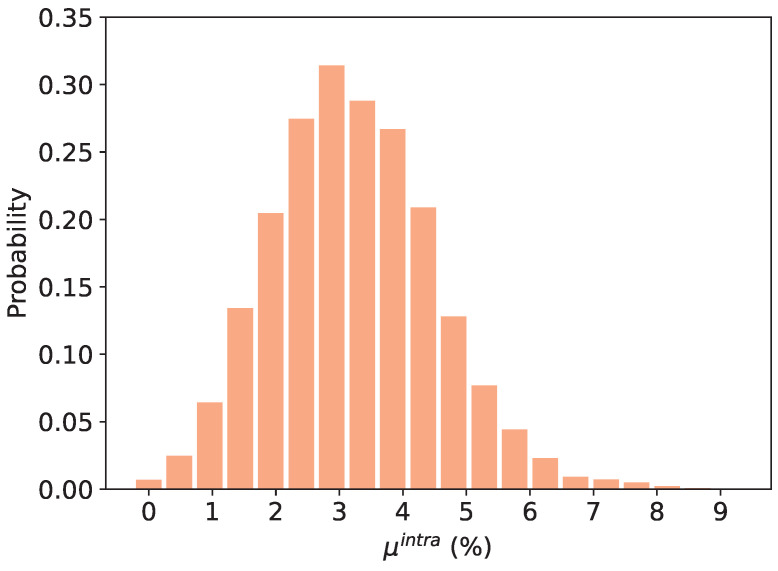
$\mu^{\mathrm{intra}}$’s of 6000 configurations with $\mu^{\mathrm{inter}}>49\%$.

Then, a selection of some oscillators with good uniqueness was made and they were studied to verify if they also exhibited good reproducibility. For this, a set of 6000 configurations with $\mu^{\mathrm{inter}}\geq 49\%$ were selected from the set of 72,000 configurations. Then, the bias of the selected configurations was measured 20 times in 20 FPGAs, and the average intra-*HD* ($\mu^{\mathrm{intra}}=\sum_{i=1}^{k=40}\mu_{\mathrm{device}\;i}^{\mathrm{intra}}$) was calculated for each configuration. The obtained histogram is shown in Figure 6. As can be seen, the majority of configurations with high uniqueness also present good reproducibility and $\mu^{\mathrm{intra}}<5\%$. Furthermore, there is a certain number of configurations with $\mu^{\mathrm{intra}}<1\%$, i.e., a low *EER*.

#### 4.1.3. Identifiability

**Figure 7:**
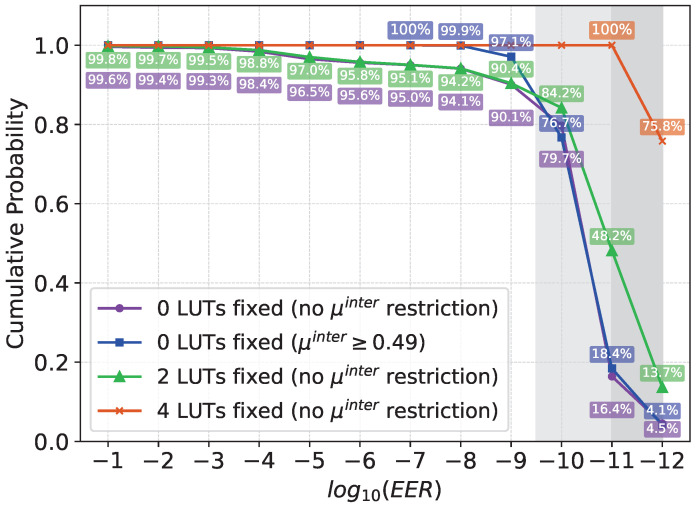
Configurations with *EER* using GenGAROs without fixed LUTs (purple), without fixed LUTs but $\mu^{\mathrm{inter}}\geq 0.49$ (blue), two fixed LUTs (green), and four fixed LUTs (orange).

In Figure 7, the *EER* obtained for the same set of 6000 configurations was found (blue). As can be seen, all configurations present a good identifiability, with *EER*s $≲10^{-7}$. Furthermore, there are some configurations with *EERs* $∼10^{-12}$, i.e., with very good identifiability. The curve obtained without applying any restriction to $\mu^{\mathrm{inter}}$ is presented in purple.

**Figure 8:**
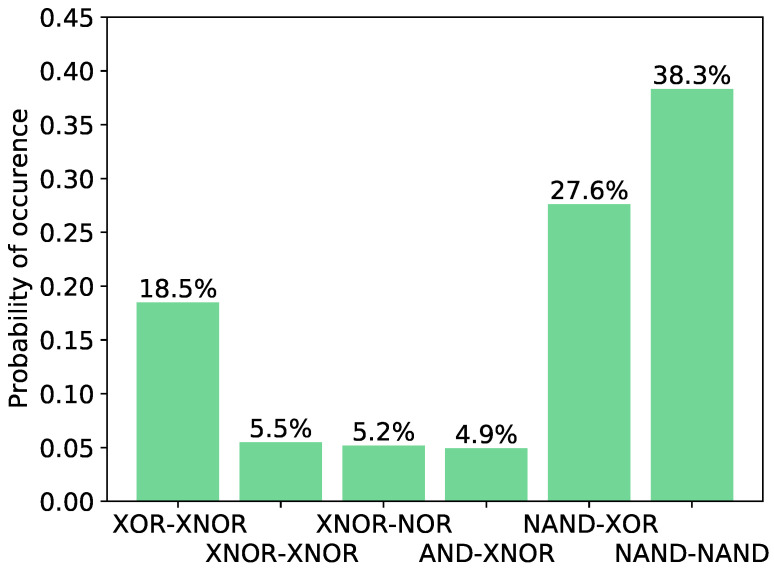
Number of configurations with *EER* $∼10^{-12}$.

As previously seen, the last two LUTs of the oscillators are crucial to obtain a PUF with high identifiability. To determine which combinations of logic operations lead to a PUF with a low *EER*, the number of configurations with *EER* $∼O(10^{-12})$ depending on the logic operations of the last two LUTs is represented in Figure 8. These combinations of logic operations do not appear to correspond to configurations with *EERs* $≳10^{-12}$. Furthermore, the number of configurations with high identifiability was normalized with respect to the initial number of configurations, with the corresponding combination of logic gates selected. As can be seen, there are two combinations of logic operations in {LUT_10_, LUT_11_} that most contribute to the set of oscillators with best identifiability: {NAND, XOR} and {NAND, NAND}. For simplicity, from this point onward, only identifiability analyses are shown; a PUF with a low *EER* implies a PUF with high reproducibility and uniqueness.

### 4.2. Restrictions to Two LUTs

Once it was found that the configurations which lead to a PUF with high identifiability tend to present these combinations of logic operations in the last two positions, the identifiability analysis was repeated using only configurations presenting these restrictions in LUT_10_ and LUT_11_. In Figure 7, the curve with the *EER*s obtained for all the selected configurations with these restrictions is shown (green). As can be seen, 84% of all the configurations present an $\mathrm{EER}≲10^{-10}$, 48% an $\mathrm{EER}≲10^{-11}$, and 14% an $\mathrm{EER}≲10^{-12}$, i.e., the majority of configurations present a high identifiability. Then, a set of 6000 configurations with restrictions over the last two LUTs was randomly generated, and the responses of the PUF instances in 40 FPGAs were analyzed. The pattern of the oscillators is analyzed in Table 2:

- Two LUTs fixed: By fixing {${\mathrm{LUT}}_{10}$, ${\mathrm{LUT}}_{11}$} with {NAND,NAND} or {NAND,XOR} gates, in both cases the best combinations tend to present a NOT gate in ${\mathrm{LUT}}_{9}$. This restrictions were applied to ${\mathrm{LUT}}_{9}$ and the analysis was repeated.
- Three LUTs fixed: By fixing the last three LUTs to the combination of logic gates NOT, NAND, NAND, or NOT, NAND, XOR, the best combinations tend to present an OR gate in ${\mathrm{LUT}}_{8}$. This restriction was applied to ${\mathrm{LUT}}_{8}$ and the analysis was repeated.
- Four LUTs fixed: If the last four LUTs are fixed to OR, NOT, NAND, NAND, or OR, NOT, NAND, XOR, a clear pattern in the rest of LUTs of the oscillators is not observed. In this way, all the generated configurations can be used to construct a PUF with high identifiability.

### 4.3. Restrictions to Four LUTs

**Figure 9:**
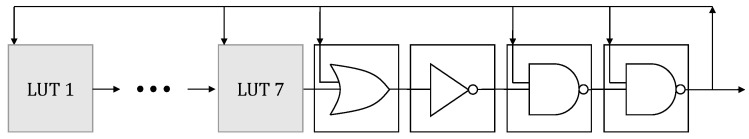
Optimal GenGAROs used to construct a GenGARO-PUF with high identifiability with OR-NOT-NAND-NAND restriction.

**Figure 10:**
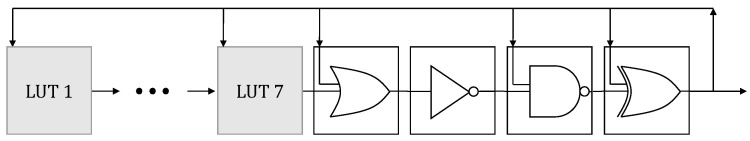
Optimal GenGAROs used to construct a GenGARO-PUF with high identifiability with OR-NOT-NAND-XOR restriction.

Finally, a set of 6000 random configurations with {OR,NOT,NAND,NAND} and {OR,NOT,NAND,XOR} gates (Figure 9 and Figure 10) in the last four LUTs was generated. Again, 20 measurements of the bias of all oscillators were measured in 40 FPGAs and the *EER* was calculated.

**Table 2:** Percentage of logic operations (%) in each position depending on the applied restrictions.

Restrictions				Gate	Number of Logic Gates in Each LUT of GenGAROs (%)										
LUT8	LUT9	LUT10	LUT11		LUT1	LUT2	LUT3	LUT4	LUT5	LUT6	LUT7	LUT8	LUT9	LUT10	LUT11
-	-	NAND	NAND	DEL	48.0	14.9	13.0	12.6	13.3	14.9	13.3	11.2	2.5	0.0	0.0
				NOT	52.0	11.7	12.8	15.1	15.1	12.8	12.4	16.2	36.8	0.0	0.0
				XOR	0.0	14.2	12.8	10.1	11.7	11.2	10.3	8.9	14.4	0.0	0.0
				XNOR	0.0	11.4	11.7	11.7	12.8	13.7	6.2	9.4	8.5	0.0	0.0
				AND	0.0	13.0	12.8	13.7	10.5	13.7	12.6	9.6	0.0	0.0	0.0
				NAND	0.0	11.4	11.9	11.9	11.9	13.0	16.5	5.5	12.1	100	100
				OR	0.0	10.3	13.5	10.3	11.2	9.4	16.7	24.5	0.0	0.0	0.0
				NOR	0.0	13.0	11.4	14.6	13.5	11.2	12.1	14.6	25.6	0.0	0.0
-	-	NAND	XOR	DEL	49.0	15.4	10.6	10.4	12.3	12.0	12.0	7.0	0.3	0.0	0.0
				NOT	51.0	17.6	10.9	14.3	14.6	11.8	9.2	16.2	47.9	0.0	0.0
				XOR	0.0	10.6	12.9	14.8	12.6	14.0	11.5	8.7	6.7	0.0	100
				XNOR	0.0	10.6	12.9	12.3	13.2	14.8	11.2	6.7	8.1	0.0	0.0
				AND	0.0	11.8	14.6	12.9	14.3	14.6	15.7	9.8	0.0	0.0	0.0
				NAND	0.0	11.2	13.2	10.1	13.4	12.3	11.5	5.9	12.0	100	0.0
				OR	0.0	11.5	14.8	12.6	12.3	11.8	16.0	25.2	0.0	0.0	0.0
				NOR	0.0	11.2	10.1	12.6	7.3	8.7	12.9	20.4	24.9	0.0	0.0
-	NOT	NAND		DEL	47.0	15.6	12.4	10.2	11.2	11.2	11.0	10.2	0.0	0.0	0.0
				NOT	53.0	13.4	14.2	15.1	14.2	12.9	13.2	12.5	100	0.0	0.0
				XOR	0.0	11.7	11.9	13.4	12.4	13.4	13.4	13.4	0.0	0.0	50.8
			NAND	XNOR	0.0	11.5	14.0	10.7	14.9	13.7	13.0	14.0	0.0	0.0	0.0
			XOR	AND	0.0	12.4	12.0	13.5	12.4	16.1	12.4	14.2	0.0	0.0	0.0
				NAND	0.0	11.0	13.4	12.7	12.2	14.4	12.2	11.7	0.0	100	49.2
				OR	0.0	11.0	10.9	11.2	11.5	9.9	11.4	23.9	0.0	0.0	0.0
				NOR	0.0	13.4	11.2	13.2	11.2	8.5	13.4	0.0	0.0	0.0	0.0
OR	NOT	NAND		DEL	44.0	14.0	13.3	8.4	12.6	10.5	13.3	0.0	0.0	0.0	0.0
				NOT	56.0	13.3	9.8	15.4	18.9	16.8	11.2	0.0	100	0.0	0.0
				XOR	0.0	9.8	11.9	14.7	7.7	14.0	15.4	0.0	0.0	0.0	49.0
			NAND	XNOR	0.0	16.8	8.4	8.4	15.4	11.2	11.9	0.0	0.0	0.0	0.0
			XOR	AND	0.0	15.4	14.7	13.3	12.6	11.9	14.7	0.0	0.0	0.0	0.0
				NAND	0.0	8.4	16.8	12.6	9.1	14.7	10.5	0.0	0.0	100	51.0
				OR	0.0	8.4	12.6	13.3	14.7	12.6	10.5	100	0.0	0.0	0.0
				NOR	0.0	14.0	12.6	14.0	9.1	8.4	12.6	0.0	0.0	0.0	0.0

As can be seen in Figure 7, 24.2% of the generated configurations present an *EER* $∼10^{-11}$, while for 75.8% the *EER* $∼10^{-12}$, using 100-bit responses. Furthermore, all configurations present $\mu^{\mathrm{intra}}$ ranging from 1.64% to 2.37%, and an extremely high $\mu^{\mathrm{inter}}$ of 49.56% and 50.13% in the worst cases. Applying these restrictions, all configurations of GenGAROs can be used to construct a GenGARO-PUF with high identifiability.

### 4.4. Comparison with Other RO-Based PUFs

In Table 3, a comparison of the GenGARO-PUF with the proposed constraints to other state-of-the-art RO-based PUFs is shown. As observed, this proposal offers high reproducibility while also exhibiting the best uniqueness.

## 5. Conclusions

An experimental study of GenGAROs has been performed to identify which configurations of logical operations exhibit better properties to construct a PUF with high identifiability. These oscillators are of particular interest, as they include some previously proposed types of oscillators as well as other new types. Several restrictions have been applied to the logic operations of the GenGAROs.

First, it has been observed that the logical operations implemented in the last LUTs of the oscillators are critical for constructing a PUF with good properties. It has been observed that without applying any restrictions to the logical operations of the oscillators, only 18.4% of the oscillators exhibit an equal error rate on the order of $10^{-11}$ or lower. However, by fixing the last two logic gates of the oscillators, this percentage increases to 48.2%. Finally, it has been observed that by applying restrictions to the last four LUTs of the GenGAROs, 100% of the generated configurations can be used to construct a PUF with an *EER* lower than $10^{-11}$ using 100-bit responses.

Following the design methodology proposed in this work, multiple GenGARO configurations can be generated and used as primitives to construct PUFs with high identifiability. These constructed PUFs improve the properties of other state-of-the-art PUFs, particularly in terms of uniqueness, identifiability, and spatial correlation of the bias of the oscillators within the FPGA. Furthermore, the significant low equal error rates obtained using these architectures makes these RO-PUF primitives promising candidates for future security applications, particularly for authentication of sensor nodes in WSNs. This approach offers valuable potential for other designers to develop novel RO-based PUFs with enhanced identifiability, contributing to creating more efficient and secure device authentication systems.

Future lines of research would include using these oscillators to construct a strong GenGARO-PUF.

## Figures and Tables

**Table 1:** Percentage of configurations (%) in each position and interval using GenGAROs without restrictions.

$\mu^{\mathrm{inter}}$ (%)	Gate	Number of Logic Gates in Each LUT of GenGAROs (%)										
		LUT1	LUT2	LUT3	LUT4	LUT5	LUT6	LUT7	LUT8	LUT9	LUT10	LUT11
[0, 1)	DEL	48.2	13.2	12.2	12.2	12.4	13.2	13.7	11.9	11.5	0.1	7.2
	NOT	51.8	12.7	11.7	13.9	13.3	12.7	14.4	16.9	19.8	77.8	0.0
	XOR	0.0	13.4	13.3	13.3	12.4	13.3	11.8	11.9	10.8	2.3	3.0
	XNOR	0.0	12.4	12.9	12.9	12.0	13.0	12.1	12.1	10.6	3.9	0.0
	AND	0.0	12.6	13.0	12.3	12.8	12.9	12.6	12.6	15.9	11.0	0.0
	NAND	0.0	12.8	13.1	12.1	12.7	11.8	14.0	12.1	11.9	1.7	89.8
	OR	0.0	11.6	12.7	11.0	12.8	11.4	10.1	10.0	1.3	0.0	0.0
	NOR	0.0	11.3	11.2	12.2	11.6	11.7	11.2	12.6	18.3	3.3	0.0
[1, 30)	DEL	46.4	12.9	12.5	12.8	13.4	12.7	13.0	13.6	17.2	15.0	74.6
	NOT	53.6	12.8	12.8	12.6	12.8	12.8	12.6	14.4	15.6	12.8	0.0
	XOR	0.0	12.9	12.9	13.2	12.9	13.2	13.1	12.6	11.6	15.5	5.8
	XNOR	0.0	13.0	12.5	13.4	12.6	12.5	12.7	12.8	11.7	15.7	10.8
	AND	0.0	12.2	12.4	11.9	11.6	12.0	12.5	10.9	14.1	2.7	0.0
	NAND	0.0	12.1	12.8	11.9	12.7	12.3	12.3	12.4	8.9	19.4	6.3
	OR	0.0	12.5	11.8	12.5	11.7	12.3	11.4	10.2	11.5	0.7	0.0
	NOR	0.0	11.7	12.4	11.7	12.3	12.2	12.4	13.0	9.4	18.2	2.5
[30, 45)	DEL	45.0	12.8	12.8	13.6	13.2	13.0	13.5	14.9	20.0	15.9	0.1
	NOT	55.0	12.8	12.8	13.0	13.2	13.2	15.0	15.7	13.1	41.2	0.0
	XOR	0.0	12.3	12.6	13.2	13.4	12.4	12.7	13.0	14.9	9.5	32.6
	XNOR	0.0	13.3	13.1	12.5	13.3	13.0	13.1	12.3	13.8	9.0	17.9
	AND	0.0	12.0	12.0	11.6	11.7	11.9	11.6	8.9	7.2	3.6	0.0
	NAND	0.0	12.7	11.8	12.5	11.8	12.4	12.4	13.3	12.6	8.0	10.9
	OR	0.0	12.4	12.7	11.6	11.8	11.6	10.4	8.9	8.6	8.3	0.0
	NOR	0.0	11.7	12.1	12.0	11.5	12.5	11.6	13.1	9.9	4.6	38.5
[45, 50]	DEL	47.7	12.8	12.7	12.5	12.9	12.6	12.8	12.3	11.2	12.8	0.0
	NOT	52.3	12.7	12.3	12.7	12.6	12.6	12.4	11.6	11.6	5.7	18.0
	XOR	0.0	12.9	12.9	12.6	12.6	12.9	12.8	12.7	12.8	13.6	12.3
	XNOR	0.0	12.4	12.4	13.0	12.8	12.9	12.8	12.8	13.1	12.9	13.5
	AND	0.0	12.2	12.7	12.2	12.3	12.3	12.3	12.9	12.5	19.0	0.0
	NAND	0.0	12.4	12.2	12.5	12.4	12.1	12.4	12.3	12.2	7.5	26.7
	OR	0.0	12.3	12.3	12.1	12.1	12.4	12.4	13.4	14.0	20.5	0.0
	NOR	0.0	12.3	12.5	12.4	12.4	12.3	12.1	12.1	12.6	8.0	29.5

**Table 3:** Comparison with other RO-based PUFs.

PUF	Year	Technology	Uniqueness	Reproducibility
GenGARO	2024	Artix 7	49.6–50.1%	1.64–2.37%
CRO [10]	2021	Spartan	49.13%	1.13%
GARO [14]	2020	Artix 7	39.1%	1.98%
TERO [11]	2018	Spartan 6	48.5%	2.6%
RO [11]	2018	Spartan 6	55.5%	2.5%

## Data Availability

The data that support the findings of this study are available from the corresponding author upon reasonable request.

## Abbreviations

The following abbreviations are used in this manuscript:

EER	Equal error rate
FAR	False acceptance rate
FF	Flip-flop
FPGA	Field-programmable gate array
FRR	False rejection rate
GARO	Galois ring oscillator
HD	Hamming distance
IoT	Internet of Things
LUT	Look-up table
MUX	Multiplexer
PUF	Physically unclonable function
RO	Ring oscillator
